# Mission-critical tasks for assessing risks from vestibular and sensorimotor adaptation during space exploration

**DOI:** 10.3389/fphys.2022.1029161

**Published:** 2022-11-25

**Authors:** Gilles Clément, Sarah C. Moudy, Timothy R. Macaulay, Michael O. Bishop, Scott J. Wood

**Affiliations:** ^1^ KBR, Houston, TX, United States; ^2^ NASA Johnson Space Center, Houston, TX, United States; ^3^ Aegis Aerospace, Houston, TX, United States

**Keywords:** spaceflight, sensorimotor system, vestibular tests, functional performance, adaptation

## Abstract

To properly assess the risk induced by vestibular and sensorimotor adaptation during exploration missions, we examined how long-duration stays on the International Space Station affect functional performance after gravity transitions. Mission-critical tasks that challenge the balance and the locomotion control systems were assessed: i.e., sit-to-stand, recovery-from-fall, tandem-walk, and walk-and-turn. We assessed 19 astronauts, including 7 first-time flyers and 12 experienced flyers, before their flight, a few hours after landing, and then 1 day and 6–11 days later. Results show that adaptation to long-term weightlessness causes deficits in functional performance immediately after landing that can last for up to 1 week. No differences were observed between first-time and experienced astronaut groups. These data suggest that additional sensorimotor-based countermeasures may be necessary to maintain functional performance at preflight levels when landing on planetary surfaces after a long period in weightlessness.

## Introduction

Astronauts returning from long-duration stays on the International Space Station (ISS) frequently report disorientation, perceptual illusions, and re-entry motion sickness (Reschke et al., 2017). The operational challenges that occur when the crewmember returns to Earth’s gravity include alterations in manual control (Moore et al., 2019), inability to egress the vehicle (Reschke et al., 2020), postural imbalance (Wood et al., 2015), and impaired locomotion (Mulavara et al., 2018). These changes are most severe during and after gravity transitions, which are the most crucial times for many critical operational tasks (e.g., landing and egress); however, only limited information is available to assess how these changes affect operations (Paloski et al., 2008).

**FIGURE 1:**
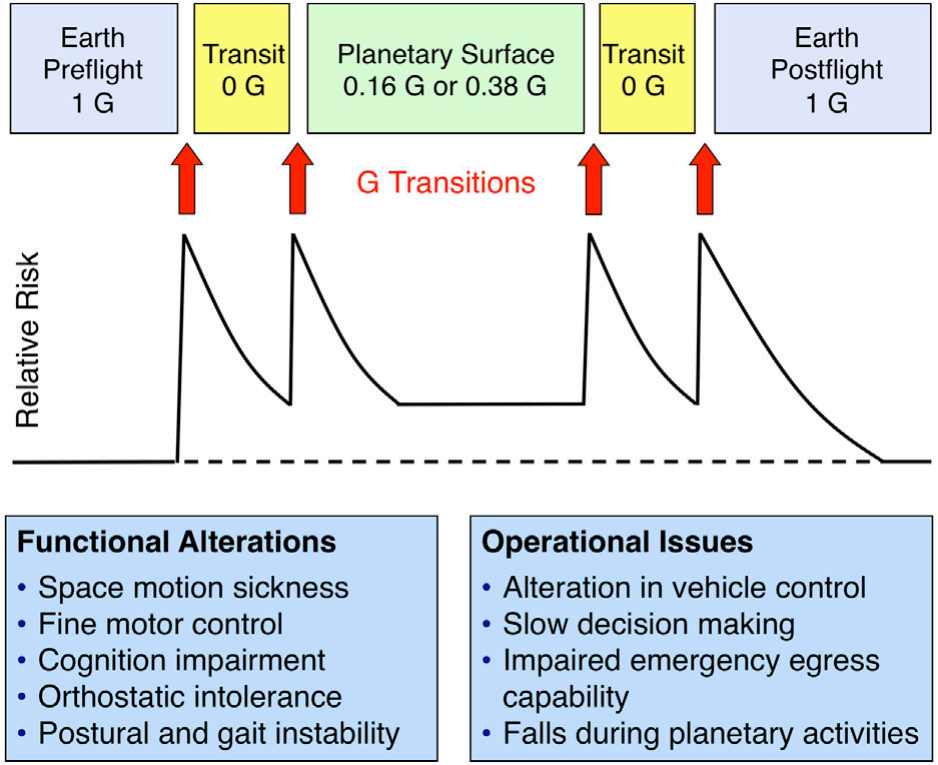
Performance decrements associated with sensorimotor disturbances occur during and after gravity (G) transitions. Photos credit NASA.

NASA’s sensorimotor exploration measures research program is assessing these operational risks. When determining the risks that could result from sensorimotor adaptation during exploration missions, it is important to consider the type and size of potential exploration mission vehicles, the amount of time spent during the transit in weightlessness (0 G) and on the planetary surface. Also critically important are how astronauts supervise and manually control the vehicle during landing and docking, and the requirements for extra-vehicular activities (EVAs) in 0 G and after landing on planetary surfaces. Human missions to the Moon and Mars will likely involve more gravity transitions and more complex docking and EVAs than low Earth orbit missions, therefore issues arising from sensorimotor adaptation may be more hazardous during these exploration missions (Figure 1).

NASA is planning human missions to the Moon with durations that range from weeks to months on the lunar surface, during which the crewmembers will transfer back and forth between the lunar surface (0.16 G) and a space station in the lunar orbit (0 G). Design reference missions for human missions to Mars include a 6-month transit in 0 G, short (30–50 days) to long (500 days) stays on the Mars surface (0.38 G), and another 6-month transit back to Earth in 0 G before landing in 1 G (Chavers et al., 2021). None of the Apollo crewmembers acknowledged spatial disorientation or locomotion problems while landing on the Moon (Paloski et al., 2008). However, the transit time to the Moon was 4.5 days during the Apollo program, which was presumably of insufficient duration for the astronauts to fully adapt to 0 G before landing on the lunar surface. Indeed, crewmembers of Space Shuttle flights lasting less than 5 days experienced no nausea and locomotion problems on return to Earth (Bacal et al., 2003).

We are currently collecting selected measures in astronauts returning from long-duration stays on the ISS to assess the risk incurred by vestibular and sensorimotor adaptation. Acquiring these selected measures, called sensorimotor standard measures for exploration, requires minimal time and resources. The most demanding mission-critical tasks that will be required during operations on a planetary surface and after return to Earth (Paloski et al., 2008) have guided the design of 4 individual functional tests: sit-to-stand, recovery-from-fall, tandem-walk, and walk-and-turn. The objective of this study is to evaluate how subjects perform functional tasks that will be required for the success of future exploration space missions and to develop countermeasures to mitigate the effects of sensorimotor deficits that could impede performance of these tasks.

## Materials and methods

### Participants

Nineteen healthy crewmembers (12 males, 7 females; mean age = 48.1 years, SD = 7.0 years) who flew on the ISS participated in this study. Seven crewmembers were flying for the first time in space, whereas 12 crewmembers had already stayed in space for one or several missions of 6 months. All subjects passed a flight physical medical examination and had no known history of vestibular or oculomotor abnormalities. The test procedures were approved by the European Space Agency Medical Board and the NASA Institutional Review Board and were performed in accordance with the ethical standards outlined in the 1964 Declaration of Helsinki. All subjects provided a written informed consent before participating in the study. Subjects provided consent for publication of identifying information and images for an online open-access publication.

Four functional tests were administered: sit-to-stand, recovery-from-fall, tandem-walk, and walk-and-turn. These tests were performed before and after 6–8-month spaceflights (mean = 198 days, SD = 70 days). A familiarization test session was conducted 239 days before launch (SD = 129 days) and a data collection session was conducted 121 days before launch (SD = 58). Tests were performed approximately 2 h after the astronauts returned to Earth, and 1 day (SD = 0.5) and 8 days (SD = 1.6) later. During each test, subjects wore a triaxial inertial measurement unit (IMU) (Opal V2 or Emerald, APDM Inc., Portland, OR, United States) attached to their trunk using an elastic band.

### Sit-to-stand

**FIGURE 2:**
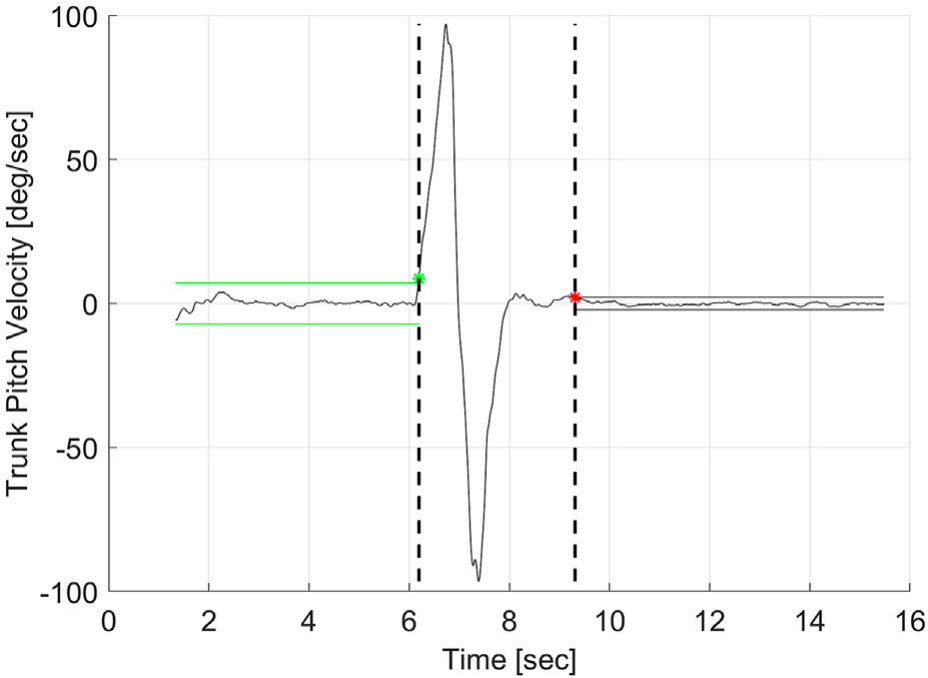
Schematic illustrating the calculation of the start and end of the sit-to-stand task based on the trunk pitch velocity.

Subjects were requested to rise as quickly as possible from a seated position without using their hands and to maintain a quiet stance for 10 s. Two trials were performed. The time elapse between the command to stand and the achievement of a stable posture was used as the measure of performance. The IMU data were used to determine when stable posture was achieved. The start and end of the stand were determined using the absolute angular trunk pitch velocity. The initial sitting period was used as a baseline. The start of the stand was defined as the first time point above 5 times the standard deviation of this baseline (Figure 2). Another baseline was calculated during the quiet stance occurring in the last 2 s of the 10-s stand period. The end of stand was defined as the first time point below the threshold of 5 times the standard deviation of this second baseline and continued to remain below this threshold for 1 s. All data were plotted and manually checked for accuracy.

### Recovery-from-fall

Subjects lay prone for 2 min and were then requested to rise as quickly as possible and maintain a quiet stance for 3.5 min (Lee et al., 2020). Only one trial was performed due to time constraints. The time elapse between the command to stand and the achievement of a stable posture was used as the measure of performance, and the same IMU and methods were used as in the sit-to-stand test.

### Tandem-walk

The tandem-walk test is used in clinics to assess control of dynamic balance in patients who may be at risk for falls (Cohen et al., 2012). Subjects were instructed to walk 10 heel-to-toe steps with their arms folded across their chests and their eyes closed (2 trials) or open (2 trials). Each trial was recorded by video. Three reviewers independently examined the videos to determine the number of correct steps during each trial. A ‘‘misstep’’ was defined as any of the following: 1) the subject’s stepping foot crossing over the plant foot; 2) the subject stepping to the side before completing the step; 3) the subject’s stepping foot swinging in a wide, arcing path before stepping down; 4) a step duration greater than 3 s; or 5) a gap larger than 10 cm between the heel of the front foot and toe of the back foot when the step was completed (Miller et al., 2018; Mulavara et al., 2018). The video order was randomized to minimize reviewer bias based on their awareness of the session. After all the reviewers had completed their assessments, the median value was used to determine the percent of correct steps for each trial. A higher percent of correct steps directly relates to better performance.

### Walk-and-turn

Subjects were requested to rise from a seated position as quickly as possible without using their hands and to stand for 10 s. After 10 s, subjects were asked to walk as quickly and safely as possible straight ahead towards a cone (4 m distance), walk around the cone, return, and sit in the chair. On the way to and from the cone, subjects stepped over a 30-cm high obstacle. This was repeated twice. This task is an adapted version of the “timed up and go” test used for diagnosing patients with unilateral vestibular deafferentation (Reschke et al., 2020; Kim et al., 2021). Measures of performance during this task included the time lapse from sit to stand, the time required for walking, and the yaw angular velocity of the trunk while walking around the cone. This test was introduced later in our study and only 7 subjects were assessed.

The start and end of the walk were determined using the resultant acceleration from the trunk IMU. The resultant acceleration was calculated using a root sum-of-squares of the x-, y, and *z*-axis acceleration signals. The resultant acceleration during the quiet stance period before the walk was used as a baseline. The start and end of the walk were defined when the resultant acceleration from the trunk IMU was above or below, respectively, 5 times the standard deviation of this baseline.

The turn rate was calculated during the cone turn only. The mean and standard deviation of the yaw angular velocity of the trunk were calculated during the straight-line walk. The start of the cone turn was based on a threshold of 5 times this standard deviation. The end of the cone turn was defined as a greater than 165-deg turn from the position at the start of the cone turn.

### Statistical analysis

Each of the 6 measures of interest were analyzed with mixed effects (multi-level) generalized linear models, using the appropriate distributional family for each dependent variable. All analyses were conducted in R Statistical Package (v4.0.5; R Core Team, 2021).

Fixed effects included an ordinal measure of return day (represented as R+0, R+1, and R+8) and a binary variable indicating if a subject was a first-time flyer. R+0 included all the measures collected up to 24 h after landing; R+1 included measures collected between 24 and 48 h after landing, and R+8 included measures collected from 6 to 11 days after landing.

Marginal means were used to estimate mean and standard errors of the parameters and the change from baseline. Within all models, we included subject-specific random intercepts to account for the repeated measures within subjects, as well as robust standard errors to address potential heteroscedasticity. Unadjusted *p*-values are reported but given the exploratory nature of the study and the limited sample sizes, emphasis was placed on estimated effect sizes and confidence limits.

Repeated measures correlations were calculated between the measures of interest. The correlations as well as the resulting *p*-values from the test of significance were also included. *p*-values were then adjusted using a Bonferroni adjustment to account for multiple testing.

When individuals were physically unable to perform the test after flight, the most extreme value (worst score) observed in other participants was imputed to better represent the data. A data summary is included in the Supplemental Material.

## Results

Re-entry motion sickness and/or orthostatic intolerance prevented 2 of the crewmembers (10.5%) from completing any of the mission-critical tests immediately after landing. Results of the statistical analysis comparing the subjects’ measures of interest during the execution of the 4 mission-critical tasks are reported in Table 1.

**TABLE 1:** Pairwise difference between return day measures (R+0, R+1, R+8) and preflight measures, and estimated effects for first-time flyers versus experienced flyers. R+0 includes measures collected up to 24 h after landing; R+1 includes measures collected between 24 and 48 h after landing, and R+8 includes measures collected from 3 to 11 days after landing. CI: 95% confidence interval of effect (min, max); *p < 0.05 relative to preflight values; EO, Eyes Open; EC, Eyes Closed.

Measure of Interest	R+0			R+1			R+8			First-time flier		
	Diff	95% CI	*p*	Diff	95% CI	*p*	Diff	95% CI	*p*	Est. effect	95% CI	*p*
Sit-to-stand	3.49	2.59, 4.40	<0.001*	0.62	−0.28, 1.53	0.189	−0.087	−0.99, 0.82	0.853	0.44	−0.44, 0.82	0.342
Time to complete (s)												
Recovery-from-fall	9.69	8.08, 11.31	<0.001*	3.26	1.69, 4.82	<0.001*	1.08	−0.49, 2.64	0.187	1.68	−0.26, 3.62	0.110
Time to complete (s)												
Tandem-walk eyes open	−60.56	−71.83, −49.30	<0.001*	−10.57	−21.84, 0.69	0.074	−1.46	−12.73, 9.80	0.802	−8.537	−21.03, 3.96	0.200
Percent correct steps (%)												
Tandem-walk eyes closed	−64.09	−74.18, −53.99	<0.001*	−47.89	−57.87, −37.99	<0.001*	−6.59	−16.57, 3.31	0.204	−3.30	−14.53, 7.99	0.574
Percent correct steps (%)												
Walk-and-turn	19.15	13.57, 25.06	<0.001*	4.53	−1.19, 9.81	0.136	1.29	−4.43, 6.57	0.661	3.76	−1.44, 9.08	0.233
Time to complete (s)												
Walk-and-turn	−75.39	−92.27, −57.79	<0.001*	−28.97	−44.93, −12.10	0.004*	−1.93	−17.89, 14.94	0.858	−17.23	−53.45, 18.87	0.405
Turn rate (deg/s)												

**FIGURE 3:**
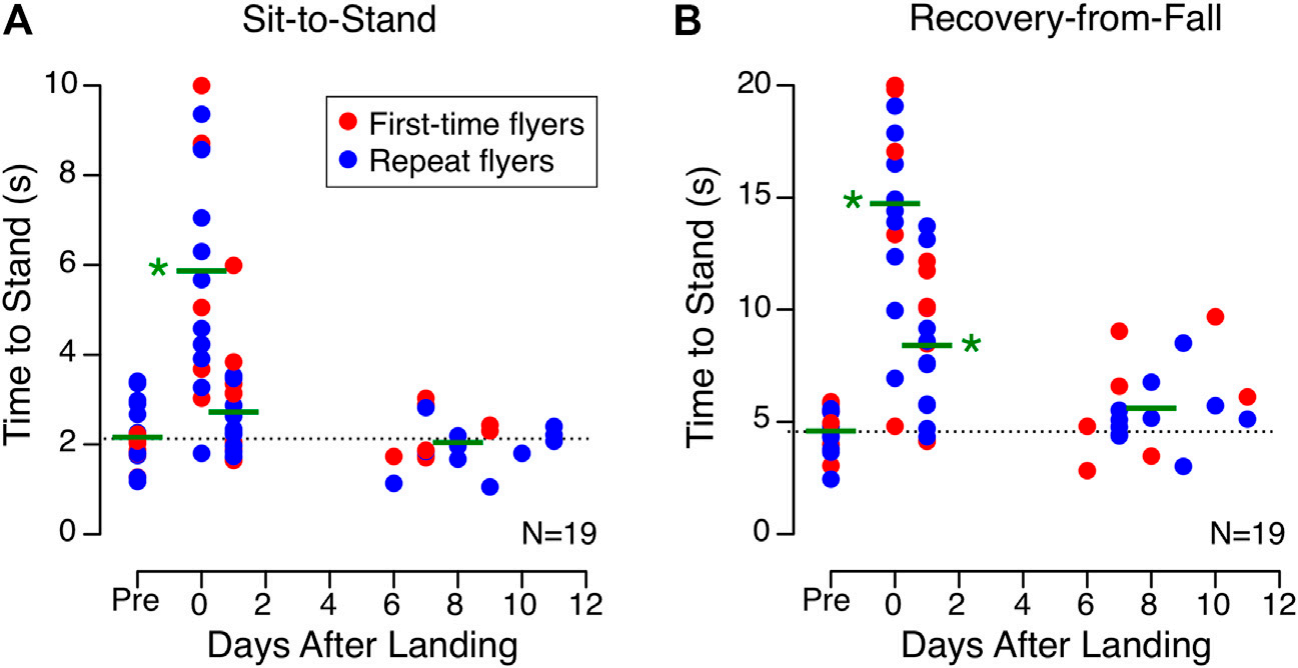
Balance tasks. Time required to assume a stable standing posture from a seated position **(A)** and a prone position **(B)** for 19 astronauts before (Pre) and after spaceflight. Each symbol represents the data from an individual subject and the horizontal blue lines represent the subjects mean for each major time point. The dotted line represents the mean of all preflight measures. *p < 0.05 relative to preflight values.

A longer time was required to assume a stable posture during the sit-to-stand test on landing day than before flight, and performance returned to baseline on R+1 (Figure 3A). The time to reach stable posture during the recovery-from-fall test was significantly longer on landing day and on R+1 than before flight (Figure 3B).

**FIGURE 4:**
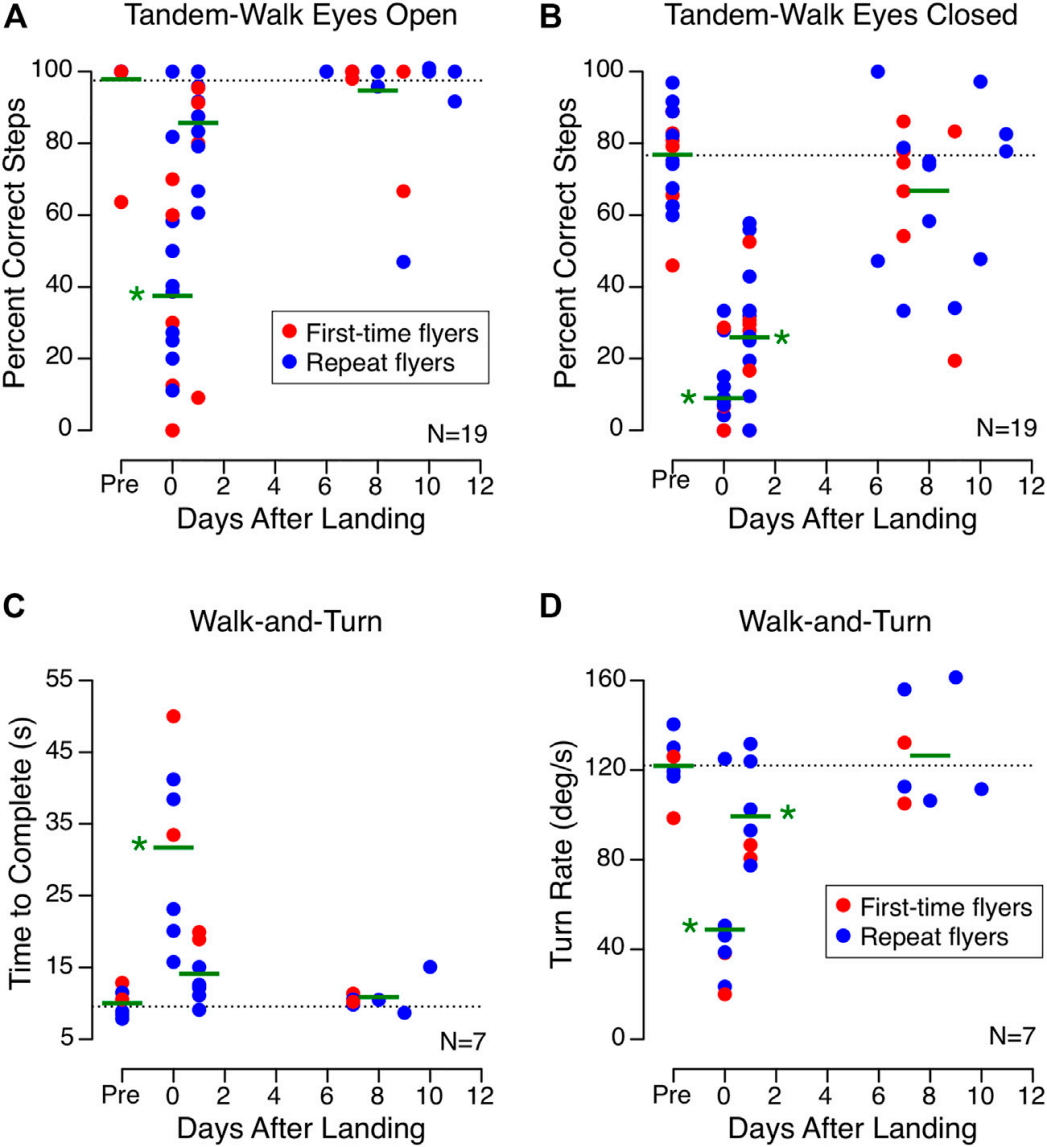
Locomotion tasks. Percent of correct steps during the tandem-walk test with eyes open **(A)** and with eyes closed **(B)** for 19 astronauts before (Pre) and after spaceflight. Time to complete the obstacle course **(C)** and turn rate around the cone **(D)** during the walk-and-turn task for 7 astronauts before (Pre) and after spaceflight. Each symbol represents the data from an individual subject and the horizontal blue lines represent the subjects mean for each major time point. The dotted line represents the mean of all preflight measures. *p < 0.05 relative to preflight values.

The percent of correct steps while performing the tandem-walk test with eyes open was significantly less on landing day than before flight, and performance returned to baseline on R+1 (Figure 4A). However, performance of this task with eyes closed was still affected on R+1 and on R+6 to R+10 in approximately half of the subjects (Figure 4B). The time required to complete the walk-and-turn test on landing day was 3 times greater than before flight, and performance returned to normal by R+1 (Figure 4C). The yaw angular velocity of the subjects’ trunks when they turned around the cone was 3 times less on landing day than before flight, and a small but significant decrease was still present on R+1 (Figure 4D).

There was no significant difference across the first-time flyers and the experienced flyers for any of the measures collected during the 4 tasks (Table 1). Significant correlations were found across all measures (Table 2).

**TABLE 2:** Repeated measures correlations matrix (Bonferroni adjusted *p*-values).

	Sit-to-stand	Recovery-from-fall	Tandem walk eyes open	Tandem walk eyes closed	Walk-and-turn time	Walk-and-turn turn rate
Sit-to-stand	1					
Recovery-from-fall	0.815 (*p* < 0.001)	1				
Tandem walk eyes open	−0793 (*p* < 0.001)	−0.783 (*p* < 0.001)	1			
Tandem walk eyes closed	−0.678 (*p* < 0.001)	−0.722 (*p* < 0.001)	0.718 (*p* < 0.001)	1		
Walk-and-turn time	0.867 (*p* < 0.001)	0.880 (*p* < 0.001)	−0.812 (*p* < 0.001)	−0.736 (*p* < 0.001)	1	
Walk-and-turn turn rate	−0.694 (*p* < 0.001)	−0.810 (*p* < 0.001)	0.756 (*p* < 0.001)	0.784 (*p* < 0.001)	−0.872 (*p* < 0.001)	1

## Discussion

This study indicates that astronauts’ balance and locomotion is significantly degraded immediately after they return from long-duration spaceflight. Balance and locomotion were still affected 1 day after landing, as shown by the significant difference in measures on R+1 relative to before flight for 1) the time required to stand during the recovery-from-fall test; 2) the number of correct steps during tandem-walk with the eyes closed; and 3) the turn rate during the walk-and-turn test (Table 1).

Previous studies of astronauts who were tested on a posture platform after short-duration spaceflight determined 2 phases of recovery: a rapid improvement of balance during the first 10–12 h, followed by a much slower recovery over the subsequent 2–4 days until preflight levels are regained (Paloski et al., 1993). Wood et al. (2015) computed the time constants of least-squares exponential recovery curves for the equilibrium scores of crewmembers performing computerized dynamic posturography in various sensory conditions after long-duration spaceflight. The time constant for the recovery of postural stability with the eyes closed after landing was reported as 96 h (4 days), which is consistent with our results.

Our results show no significant differences between the first-time flyers and experienced flyers in any of the measures collected in this study. Two recently published studies performed after long-duration ISS missions report conflicting results. On one hand, Schoenmaekers et al. (2022) studied the eye movements of 23 cosmonauts and reported a postflight decrease in ocular counter-rolling (OCR) during centrifugation that recovered faster in experienced flyers compared to first time flyers. On the other hand, Rosenberg et al. (2022) studied the posture of 29 astronauts and showed that postural instabilities were not correlated with the number of flights, mission duration, or timing on the tests across first-time and experienced flyers. Earlier studies after missions ranging from 1 to 2 weeks on board the Space Shuttle had shown that astronauts flying on their first mission exhibited significantly higher postural sway (Paloski et al., 1993) and greater alterations in the frequency spectra of pitch head movements during locomotion (Bloomberg et al., 1977; Glasauer et al., 1995) as compared to astronauts with prior spaceflight experience. Beside mission duration, a major difference between Space Shuttle and ISS missions is that astronauts were not exercising on board the Space Shuttle, whereas on the ISS they typically spend 2 h daily for 5 days a week on various exercise devices. Wood et al. (2015) showed that postural instability after long-duration ISS missions was less severe and recovered faster in those crewmembers who exercised on a resistive exercise device that involved large head and body motions, compared to an earlier system which generated less head movements.

In a follow-up of Hallgren et al. (2016) study, Schoenmaekers et al. (2022) measured a 3-deg decrease in OCR on R+3 when subjects sitting upright were exposed to a 1-g centripetal acceleration along their interaural axis on a centrifuge. These authors claim that this decrease in OCR is responsible for the alteration in gaze stabilization and postural stability after spaceflight. However, before the flight, when averaged across all subjects, directions of rotation, and eyes, the amplitude of OCR in Schoenmaekers’ study was 5.8 deg for a 45-deg tilt of the gravito-inertial vector, which corresponds to a gain of 0.13, i.e., a poorly effective compensatory reflex. On R+3, the mean OCR gain was 0.07. Obviously, a decrease in OCR gain from 0.13 to 0.07 cannot account for the large post-flight decrements in sensorimotor performance seen in our study. In addition, other studies have shown that OCR recovers to its preflight value by R+3 after both short-duration (Clément et al., 2007) and long-duration (Reschke et al., 2018) spaceflights.

A major difference between these studies and Schoenmaekers’ study is that in the latter, subjects were technically not tilted relative to gravity. When subjects are actually tilted in roll relative to gravity by 30 deg after spaceflight, they perceive a tilt of approximately 40 deg (Clément et al., 2001); when tilted by 60 deg, they perceive approximately 80 deg (Clément et al., 2007; Clément and Wood, 2013a; 2023b; Clément and Wood, 2014) etc., i.e., a 30% overestimation. Therefore, by contrast with the ∼3-deg post-flight decrease in OCR, there is an increase in the sensitivity to perceived tilt by the vestibular system. Such dissociation between eye movement and perception has been reported during various conditions where gravity is altered (Clément et al., 2019). The increase in perceived sense of tilt after spaceflight is in agreement with the results of electrophysiological (Boyle et al., 2001) and anatomical (Ross et al., 2000) studies in animals, which show a hypersensitivity of the vestibular afferents after adaptation to microgravity. We believe that this increase in otolith sensitivity is more likely responsible for the postural instabilities when standing or when walking seen in our study after spaceflight, rather than the decrease in OCR. Unfortunately, Hallgren et al. (2016) and Schoenmaekers et al. (2022) did not report on the perception of tilt (the so-called somatogravic illusion) in their subjects.

The vestibular system is more challenged when subjects stand from a prone position than from a sitting position, which might explain the slightly longer recovery time during the recovery-from-fall task. However, other physiological systems are involved when standing from a prone position, such as the initiation of cardiovascular responses for maintaining orthostatic tolerance and the activation of muscles of the arms, legs, and back muscles which act against gravity.

The time required to assume a stable posture during the sit-to-stand test and the recovery-from-fall test, as with computerized dynamic posturography, involves static posture, whereas tandem-walk and walk-and-turn are dynamic balance tasks, with the latter being more representative of an operational task that the crewmembers may be required to perform upon return to Earth or when they land on another planet. Completing these mission-critical tasks requires visual, vestibular, and proprioceptive inputs for postural control, and requires coordination of the lower limbs, and cognitive processes such as mental representation of space, navigation, and memory for locomotion control. Our results indicate that performance during the tandem-walk test and the walk-and-turn test was still impaired in some crewmembers 1 week after they returned from 6 to 8 months flights on the ISS. This result is in agreement with previous studies showing adaptive alterations in the coordination among eye, head, and trunk movement during locomotion after long-duration space flight (Bloomberg et al., 1977; Courtine and Pozzo, 2004). A task as simple as walking in a straight line or towards a target, turning around and returning needs a larger cognitive effort after spaceflight, which would slow down motor performance. This implies that mechanisms like computing self-displacement from somatosensory and vestibular inputs, and updating of spatial information are disturbed by the stay in microgravity and the gravity transitions during return to Earth, and must be re-acquired (Glasauer et al., 1995).

After an equivalent duration of exposure to a weightless environment during transit to Mars, the crewmembers will take several days to recover their nominal 1 G functional performance after they land on Mars, which could delay mission objectives (e.g., habitat set-up, EVAs, sample collection), because the astronauts’ performance would be suboptimal. However, this may be sufficient to maintain acceptable performance of functional tasks on the lunar and Martian surfaces.

The balance and locomotion tests in this study challenge vestibular function, which is paramount for efficiently completing critical mission tasks. Previous studies have shown that balance control (Wood et al., 2015; Miller et al., 2018), locomotion (Bloomberg et al., 1997; Courtine and Pozzo, 2004), spatial orientation (Clément and Wood, 2013, 2014), and navigation (Glasauer et al., 1995), as well as spatial representation (Clément et al., 2013), memory (Hitier et al., 2014), and time perception (Clément et al., 2018) are commonly altered after spaceflight. Therefore, countermeasures will be needed not only for risks explicitly related to vestibular function, but also for risks related to multi-sensory perception and control of orientation and movement, neural coordination of movement including posture and locomotion, and autonomic and emetic function.

Artificial gravity has been proposed as the ultimate countermeasure during spaceflight because it would mimic the normal gravitational environment on Earth (Clément, 2017). However, due to engineering, operational, and budgetary constraints, there is no current plan for rotating the spacecraft during the journey to and from Mars. An onboard centrifuge has been proposed as an alternative. However, intermittent centrifugation during bed rest has not proven to be beneficial for postural control (Clément et al., 2022a; 2022b). More studies are needed to determine the minimum artificial gravity level and duration needed for mitigating physiological deconditioning (Clément et al., 2019). Also, artificial gravity would not prevent the changes in sensorimotor and vestibular performance immediately following gravity transitions.

Astronauts exercise during spaceflight to protect muscle strength and function, but exercise alone cannot guarantee optimal performance of functional tasks after spaceflight. Balance training may also be needed to maintain balance control at the preflight levels during the first hours and days after flight. Other operational countermeasures include post-flight rehabilitation, sensory augmentation, and combining non-pharmacological countermeasures with new anti-motion sickness drugs administration routes. In the future, fitness for duty sensorimotor assessments tasks will be defined and these assessments will provide a quantitative index of readiness to perform key operational tasks and will validate self-administered integrative countermeasure approaches suitable for autonomous exploration missions.

No conclusive evidence exists that prolonged (months to years) exposure to weightlessness produces irreversible changes to the vestibular system. However, further characterization of potential long-term health consequences is needed because so far only a dozen individuals have flown beyond 8 months. Anatomical changes have been detected in the vestibular sensory epithelia of animals after they flew for several weeks or longer in space (Ross, 2000; Boyle, 2001). However, the functional significance of these changes is unclear. Radiation exposure is known to impact some areas of the central vestibular system, such as the hippocampus, and imaging studies have revealed alterations in functional brain connectivity after long-duration spaceflight (Pechenkova et al., 2019). Validated, sensitive instruments and methods that detect early impairment of vestibular and sensorimotor functions, such as those used in the present study, will be critical for assessing the effects induced by gravity transitions and for testing countermeasures.

## Data Availability

All data from this paper are available upon request through the NASA Life Sciences Data Archive: https://nlsp.nasa.gov/explore/lsdahome/datarequest.
