# Supplementary material for: Mission-critical tasks for assessing risks from vestibular and sensorimotor adaptation during space exploration
Source: Front Physiol. 2022 Nov 25;13:1029161. doi: 10.3389/fphys.2022.1029161 (PMC9733831; doi:10.3389/fphys.2022.1029161)
Supplement: Supplementary file 1 [file Table1.DOCX]

**Data Summary**

|  | **Preflight** | | **R+0** | | **R+1** | | **R+8** | | **Overall** | |
| --- | --- | --- | --- | --- | --- | --- | --- | --- | --- | --- |
|  | **Repeat Flight (N=12)** | **First Flight (N=7)** | **Repeat Flight (N=12)** | **First Flight (N=7)** | **Repeat Flight (N=12)** | **First Flight (N=7)** | **Repeat Flight (N=12)** | **First Flight (N=7)** | **Repeat Flight (N=48)** | **First Flight (N=28)** |
| **Sit-to-Stand,**  **Time to Complete (s)** |  |  |  |  |  |  |  |  |  |  |
| Mean  (SD) | 2.33 (0.78) | 1.97 (0.45) | 5.43 (2.60) | 6.12 (3.12) | 2.48 (0.65) | 3.37 (1.44) | 1.90 (0.49) | 2.43 (0.67) | 3.05 (1.99) | 3.47 (2.34) |
| Median  [Min,  Max] | 2.26 [1.17, 3.41] | 1.87 [1.27, 2.73] | 5.13 [1.80, 9.36] | 5.32 [1.33, 9.36] | 2.30 [1.70, 3.53] | 3.35 [1.64, 5.99] | 1.90 [1.05, 2.82] | 2.43 [1.70, 3.35] | 2.26 [1.05, 9.36] | 2.80 [1.27, 9.36] |
| Missing | 1  (8.3%) | 0  (0%) | 0  (0%) | 0  (0%) | 0  (0%) | 0  (0%) | 0  (0%) | 0  (0%) | 1  (2.1%) | 0  (0%) |
| **Recovery-from-Fall,**  **Time to Complete (s)** |  |  |  |  |  |  |  |  |  |  |
| Mean  (SD) | 4.47 (1.14) | 5.05 (0.875) | 13.6 (4.99) | 15.6 (5.03) | 6.81 (2.85) | 9.75 (2.60) | 5.22 (1.86) | 6.56 (1.87) | 7.46 (4.64) | 9.01 (4.85) |
| Median  [Min,  Max] | 4.37 [2.44, 5.90] | 4.95 [3.78, 6.48] | 13.9 [4.80, 20.0] | 16.8 [5.71, 20.0] | 6.65 [3.13, 11.7] | 9.16 [5.79, 13.7] | 5.14 [2.82, 9.68] | 6.59 [4.37, 9.04] | 5.62 [2.44, 20.0] | 7.55 [3.78, 20.0] |
| Missing | 1  (8.3%) | 0  (0%) | 1  (8.3%) | 1  (14.3%) | 0  (0%) | 0  (0%) | 0  (0%) | 0  (0%) | 2  (4.2%) | 1  (3.6%) |
| **Tandem Walk Eyes Open, Percent Correct Steps (%)** |  |  |  |  |  |  |  |  |  |  |
| Mean  (SD) | 100  (0) | 90.0 (17.0) | 41.9 (28.7) | 25.1 (29.9) | 88.7 (13.8) | 80.5 (32.3) | 94.5 (15.2) | 95.2 (12.6) | 80.9 (29.1) | 72.7 (36.6) |
| Median  [Min,  Max] | 100 [100, 100] | 100 [63.6,  100] | 39.5  [0,  100] | 12.5  [0,  70.0] | 93.8 [60.6, 100] | 91.3 [9.09, 100] | 100 [47.0, 100] | 100 [66.7, 100] | 100  [0,  100] | 93.4  [0,  100] |
| Missing | 1  (8.3%) | 0  (0%) | 0  (0%) | 0  (0%) | 0  (0%) | 0  (0%) | 0  (0%) | 0  (0%) | 1  (2.1%) | 0  (0%) |
| **Tandem-Walk Eyes Closed, Percent Correct Steps (%)** |  |  |  |  |  |  |  |  |  |  |
| Mean  (SD) | 77.3 (13.2) | 69.2 (21.3) | 10.6 (11.1) | 9.28 (13.8) | 27.9 (19.0) | 22.6 (18.7) | 67.2 (22.7) | 67.5 (24.3) | 45.8 (32.1) | 42.1 (33.0) |
| Median  [Min,  Max] | 75.2 [59.9, 97.0] | 79.2 [35.2, 88.9] | 8.33  [0,  33.3] | 0  [0,  29.6] | 29.0  [0,  57.8] | 27.8  [0,  52.5] | 74.5 [33.3, 100] | 74.6 [19.4, 87.9] | 45.1  [0,  100] | 32.9  [0,  88.9] |
| Missing | 1  (8.3%) | 0  (0%) | 1  (8.3%) | 0  (0%) | 0  (0%) | 0  (0%) | 0  (0%) | 0  (0%) | 2  (4.2%) | 0  (0%) |
| **Walk-and-Turn,**  **Time to Complete (s)** |  |  |  |  |  |  |  |  |  |  |
| Mean  (SD) | 9.16 (1.62) | 11.7 (1.64) | 27.7 (11.4) | 33.5 (NA) | 12.0 (2.18) | 19.4 (0.713) | 10.9 (2.44) | 10.8 (0.801) | 15.2 (9.55) | 16.7 (8.37) |
| Median  [Min,  Max] | 8.64 [7.84, 11.5] | 11.7 [10.5, 12.9] | 23.1 [15.8, 41.2] | 33.5 [33.5, 33.5] | 12.2 [9.08, 15.0] | 19.4 [18.9, 19.9] | 10.5 [8.67, 15.1] | 10.8 [10.2, 11.3] | 11.5 [7.84, 41.2] | 12.9 [10.2, 33.5] |
| Missing | 8 (66.7%) | 5 (71.4%) | 7 (58.3%) | 6 (85.7%) | 7 (58.3%) | 5 (71.4%) | 7 (58.3%) | 5 (71.4%) | 29 (60.4%) | 21 (75.0%) |
| **Walk-and-Turn,**  **Turn Rate (deg/s)** |  |  |  |  |  |  |  |  |  |  |
| Mean  (SD) | 127 (10.7) | 112 (19.4) | 56.8 (39.5) | 38.3 (NA) | 106 (22.2) | 83.6 (4.16) | 130 (26.7) | 119 (19.2) | 104 (39.4) | 95.4 (31.5) |
| Median  [Min,  Max] | 125 [117, 140] | 112 [98.6, 126] | 46.2 [23.4, 125] | 38.3 [38.3, 38.3] | 102 [77.4, 132] | 83.6 [80.7, 86.6] | 113 [106, 161] | 119 [105, 132] | 113 [23.4, 161] | 98.6 [38.3, 132] |
| Missing | 8 (66.7%) | 5 (71.4%) | 7 (58.3%) | 6 (85.7%) | 7 (58.3%) | 5 (71.4%) | 7 (58.3%) | 5 (71.4%) | 29 (60.4%) | 21 (75.0%) |
